# What Can Machine Learning Approaches in Genomics Tell Us about the Molecular Basis of Amyotrophic Lateral Sclerosis?

**DOI:** 10.3390/jpm10040247

**Published:** 2020-11-26

**Authors:** Christina Vasilopoulou, Andrew P. Morris, George Giannakopoulos, Stephanie Duguez, William Duddy

**Affiliations:** 1 Northern Ireland Centre for Stratified Medicine, Altnagelvin Hospital Campus, Ulster University, Londonderry BT47 6SB, UK; Vasilopoulou-C@ulster.ac.uk (C.V.); s.duguez@ulster.ac.uk (S.D.); 2 Centre for Genetics and Genomics Versus Arthritis, Centre for Musculoskeletal Research, Manchester Academic Health Science Centre, University of Manchester, Manchester M13 9PT, UK; andrew.morris-5@manchester.ac.uk; 3 Institute of Informatics and Telecommunications, NCSR Demokritos, 153 10 Aghia Paraskevi, Greece; ggianna@iit.demokritos.gr; 4 Science For You (SciFY) PNPC, TEPA Lefkippos-NCSR Demokritos, 27, Neapoleos, 153 41 Ag. Paraskevi, Greece

**Keywords:** Amyotrophic Lateral Sclerosis, machine learning, genome-wide association studies, GWAS, genomics, ALS pathology, gene prioritization

## Abstract

Amyotrophic Lateral Sclerosis (ALS) is the most common late-onset motor neuron disorder, but our current knowledge of the molecular mechanisms and pathways underlying this disease remain elusive. This review (1) systematically identifies machine learning studies aimed at the understanding of the genetic architecture of ALS, (2) outlines the main challenges faced and compares the different approaches that have been used to confront them, and (3) compares the experimental designs and results produced by those approaches and describes their reproducibility in terms of biological results and the performances of the machine learning models. The majority of the collected studies incorporated prior knowledge of ALS into their feature selection approaches, and trained their machine learning models using genomic data combined with other types of mined knowledge including functional associations, protein-protein interactions, disease/tissue-specific information, epigenetic data, and known ALS phenotype-genotype associations. The importance of incorporating gene-gene interactions and cis-regulatory elements into the experimental design of future ALS machine learning studies is highlighted. Lastly, it is suggested that future advances in the genomic and machine learning fields will bring about a better understanding of ALS genetic architecture, and enable improved personalized approaches to this and other devastating and complex diseases.

## 1. Introduction

Amyotrophic Lateral Sclerosis (ALS) is a progressively fatal, late-onset motor neuron disorder that is predominately characterised by the loss of upper and lower motor neurons. Progressive muscle atrophy in ALS patients leads to swallowing difficulties, paralysis and ultimately to death from neuromuscular respiratory failure [1,2,3]. ALS is the most common type of motor neuron disorder, and has peak onset at 54–67 years old, although it can affect individuals of any age [2,3,4,5]. Patients typically survive 2–5 years after the first symptoms occur, with 5–10% surviving more than 10 years [1,2,6]. A population-based study of estimated ALS incidence in 10 countries found that prevalence could increase more than 31% from 2015 to 2040 [4]. Thus, there is an increasing need to understand ALS pathology and the molecular pathways involved, towards prevention or successful therapeutic intervention.

There are two major classifications among ALS patients, based on family history: 5–10% of cases are genetically linked, and are classified as *familial*, having one or more relatives that suffer from ALS, while 90% are classified as *sporadic*, in which a familial history is not established, and where a genetic cause is usually not identified [7]. However, the distinction between the two categories is not always simple, with familial ALS-associated mutations also being present among sporadic ALS cases [3]. The extent and form of genetic contribution to sporadic ALS remains unclear, but genetic factors are considered to play an important role in the disease pathology [3,8]. Further investigation of the genetic architecture of both familial and sporadic cases is necessary.

In recent years, advances in high-throughput technologies have enabled the discovery of multiple Single Nucleotide Polymorphisms (SNPs) that are associated with ALS, mainly by the application of the Genome-Wide Association Study (GWAS) approach. GWAS aims to identify SNPs and other types of genetic variation (such as structural variants, copy number variations and multiple nucleotide polymorphisms) that are more frequent in patients than in people without the disease [9]. Statistical tests are carried out for disease association across genetic markers numbering from hundreds of thousands up to millions, depending on the genomic analytical platform. The most popular genotype-phenotype association studies use statistical models such as logistic or linear regression, depending on whether the trait is binary (i.e., case-control studies, such as ALS versus healthy controls) or quantitative (e.g., different scales of height). GWAS has been successful in discovering tens of thousands of significant genotype-phenotype associations in a large spectrum of diseases and traits, such as schizophrenia, anorexia nervosa, body-mass index (BMI), type 2 diabetes, and ALS [10,11,12,13]. Over the past decade, the discovery of significant genotype-phenotype associations has provided new insights into disease susceptibility, pathology, prevention, drug design and personalized medical approaches [11,14,15].

Rapid recent technological advances and great efforts in the field have led to the genomic profiling of large ALS cohorts, providing new insights into the pathology of ALS [12]. Initiatives such as Project MinE and dbGaP have contributed to the systematic release of ALS GWAS data [16,17]. The ALSoD publicly available database for genes that are implicated in ALS records 126 genes, with a subset having been reproduced in multiple studies [18]. As of July 2020, the GWAS catalogue has published 317 variants and risk allele associations with ALS [10].

The scope of this review covers genome-wide association studies that employ machine learning approaches with the aim to understand ALS pathology through gene prioritization. A search of PubMed and Google Scholar for the terms “amyotrophic lateral sclerosis”, “GWAS” and “machine learning” yielded 420 results, of which 7 research papers were identified as falling into this scope. Machine learning studies that refer to the estimation of ALS heritability, to drug repurposing/prediction for ALS, or survival analyses, are not considered.

The review is structured as follows: first, knowledge from relevant literature about ALS pathology and genetic architecture is summarized, then the central challenges and limitations of traditional GWAS studies are introduced in the context of ALS, while the third section provides a brief overview of key machine learning concepts along with a description and comparison of published feature selection and machine learning approaches using ALS GWAS datasets. The main contribution of the review is to outline the challenges of ALS genomic studies, summarizing and comparing how these have been addressed by the collected research papers. Finally, the further use of machine learning as a method to understand ALS pathology is advocated.

### 1.1. Current Knowledge of Molecular Pathways Implicated by the Functions of Known ALS-Linked Genes

Our current knowledge of the aetiology and the genetic architecture of ALS is still elusive. Genetic mutations, environmental contributions, epigenetic changes and DNA damage are hypothesized as potential causal factors that ultimately lead to motor neuron death [19,20]. Variants in more than 30 genes are recognized as monogenic causes of ALS [12,19,21,22,23]. The most frequent monogenic cause in European populations is the intronic hexanucleotide GGGGCC (G4C2) repeat expansion (HRE) in the *C9orf72* gene [24,25]. Other genes linked to ALS with high reproducibility include Cu/Zn superoxide dismutase 1 *SOD1*, fused in sarcoma *FUS*, and transactive response DNA-binding protein of 43 kD *TARDBP/TDP-43* [19]. The discovery of risk gene mutations has helped to unravel the molecular mechanisms of ALS, and may lead ultimately to targeted therapy and stratified drug discovery [19,26,27,28].

Numerous studies have been published aimed at explaining motor neuron death, investigating the functional effects of specific mutations of known risk-associated genes such as *C9orf72, FUS, SOD1* and *TDP-43* [23,27]. Recent systematic reviews from our group have summarised the molecular pathways and biomarkers for which there are strong supporting evidence in ALS [19,26,27]. The molecular pathways affected in ALS can be grouped as follows (see [27] for detailed review):

- Mitochondrial dysfunction as a direct or indirect consequence of ALS-associated gene mutations *CHCHD10, FUS, SOD1, C9orf72 and TDP-43* can lead to an increase in oxidative stress, an increase in cytosolic calcium, ATP deficiency and/or stimulation of pro-apoptotic pathways [20,22,27,29,30].
- Oxidative stress can also be derived from a stimulation of NADPH oxidase, as observed with *ATXN2* mutations [31], or from deficiency in the elimination of Reactive Oxygen Species (ROS) as observed with some *SOD1* mutations in familial cases [27,32,33]. It then may contribute to DNA damage. Interestingly, other mutations on the ALS-associated genes *NEK1* [34], *SETX* [35] and *C21orf2* [36] are suspected to alter the DNA repair machinery, leading to an accumulation of oxidative damage over time. Consequently, these events could ultimately lead to motor neuron death [34,37].
- Disrupted axonal transport has been directly linked to a mutation in the C-terminal of the ALS-associated gene, *KIF5A* [12,38], and to mutations in genes encoding for neurofilaments (*NEFH*), microtubules and motor proteins (*PFN1*,*TUBA4A*, *DCTN1*) [27,39]. Consequently, organelle transport, protein degradation, and RNA transport are affected, disrupting cellular homeostasis. Similarly, axonal transport disruptions have been observed in fALS patients harboring mutations in non-cytoskeletal-related genes such as *SOD1* [38].
- Protein degradation is suspected to be a key pathway that is defective in ALS. This can be a direct consequence of mutations in ALS-associated genes involved in proteasome activity and the autophagy pathway, such as *UBQLN2*, *VCP*, *SQSTM1/P62*, *OPTN*, *FIG4*, *Spg11*, or *TBK1* [40], and may lead to an accumulation of misfolded and non-functional proteins [27,41]. It can also be an indirect consequence of other mutations leading to the formation of protein aggregates such as SOD1, FUS, TDP43, C9orf72-derived DPR - aggregates that in turn impair the proteasome and autophagic degradation pathways [42], thus exacerbating the accumulation of misfolded proteins. Consequently, the blockade of autophagy pathways may affect vesicle secretion [43,44]. Interestingly, some ALS-associated genes are known to be directly or indirectly involved in exosome biogenesis such as *CHMP2B* [45] or *C9orf72* [46], respectively.
- Glutamate-mediated excitotoxicity has been suggested to cause motor neuron deterioration, and could be an indirect consequence of ALS-associated gene mutations such as in *SOD1* or *C9orf72*, resulting in an elevated level of glutamate in the cerebrospinal fluid of patients [47,48,49].
- RNA processing and metabolism is another key pathway affected in ALS. For example, mutations to RNA-binding proteins encoded by *FUS*, *TDP-43*, *hnRNPA1*, *hnRNPA2B1*, and *MATR3*, result in altered mRNA splicing, RNA nucleocytoplasmic transport and translation [27,50,51,52,53,54,55], as well as in the generation and accumulation of toxic stress granules [56]. Similarly, accumulation of toxic RNA foci can be observed in motor neurons in the context of *C9orf72* mutations, and may lead to the sequestration of splicing proteins, thus affecting RNA maturation and translation [57]. The biogenesis of microRNA is also directly affected by mutated *FUS*, *TDP-43*, or *C9orf72*-mediated DPRs, thus having an impact on the expression of genes involved in motor neuron survival [27,58].

Understanding the functional processes that drive ALS pathology has proven to be a difficult and complex task, compounded by the heterogeneity that characterises the disease. The gene products of the 30 or more known ALS-associated genes interact with each other, are implicated in multiple molecular pathways, and result in multiple disease phenotypes, making functional curation and interpretation complex [19,27]. In addition, these monogenic causes in ALS occur only in ∼15% of sporadic ALS and ∼66% of familial ALS patients, so that more than 80% of the ALS population do not currently have any known ALS-associated mutations [19,21]. Nonetheless, acquiring an in-depth understanding of the molecular mechanisms and the genetic architecture of ALS could potentially lead to the identification of multiple patient strata and therefore targeted therapies to be applied to different subgroups of ALS patients.

### 1.2. The Genetic Architecture of ALS

The genetic contribution to familial and sporadic ALS has not been fully explained by genotype-phenotype discoveries [8,25], and the known Mendelian causes of ALS represent only a small proportion of the ALS population [19,21]. Nonetheless, estimates of heritability are high in sporadic ALS patients - for example, 61% in a twin meta-analysis study - suggesting that genetic factors are strongly represented in sporadic ALS and that further investigation may yet identify novel causal variants and/or multilocus interactions that could account for this high estimated heritability [59].

So far, evidence supports a model implicating rare variants (minor allele frequency <1%) along with non-genetic causes, such as environmental factors [3,36,60,61]. Large GWAS efforts suggest a genetic architecture for ALS that falls somewhere in the middle of the spectrum of genetic pathology in terms of effect size and prevalence of risk variants-i.e., an *intermediate genetic architecture*, lying between conditions such as schizophrenia which have many common variants each imparting a small increase to disease risk, and conditions such as Huntington’s disease which are caused by rare large-effect variants located in a single gene [3,20,62,63].

Many ALS-associated variants, particularly for *C9orf72*, also contribute to other conditions such as frontotemporal dementia (FTD) and cerebellar disease, suggesting that ALS is a multi-system syndrome [3,60,61]. ALS has an established overlap with other neurodegenerative and neuropsychiatric disorders, investigation of which could lead to insights into the understanding of pathology [3,5,25,60,64,65]. An example of this is the degree of overlap between familial ALS (∼40%) and familial FTD (∼25%) patients that carry the G6C4 expansion of *C9orf72* [65,66]. *C9orf72* hexanucleotide expansion has been associated to multiple traits including Alzheimer’s and Parkinson’s diseases, ataxia, chorea and schizophrenia [21,67,68,69]. A population-based GWAS study reported a higher prevalence of psychosis, suicidal behaviour, and schizophrenia, in Irish ALS kindreds, which was associated with the C9orf72 repeat expansion, based on an aggregation analysis [64]. Further evidence for a shared susceptibility to ALS was provided by the greater occurrence of dementia among first-degree relatives of ALS patients [69]. Several studies have suggested that the genetic overlap between ALS and other neurodegenerative and neuropsychiatric disorders could also be explained by the presence of ALS-associated pleiotropic variants that influence multiple, and in some cases quite distinct, phenotypic traits [70,71,72]. One study that supports this hypothesis is that of O’Brien et al., which shows that first-degree and second-degree relatives of Irish ALS patients have a significantly higher prevalence of schizophrenia and neuropsychiatric diseases than healthy controls, including obsessive-compulsive disorder, psychotic illness, and autism-the authors performed k-means clustering and calculated the relative risk to estimate aggregation [71,73,74,75].

Further investigation is needed to achieve a deep understanding of ALS heritability and genetic architecture, incorporating pleiotropic gene effects into experimental design.

## 2. ALS-Specific GWAS Challenges and Limitations

So far, numerous ALS GWAS studies have been published, aiming to identify novel ALS-associated variants through standard genotype-phenotype analyses. The first was published in 2007, providing genomic data for 276 cases and 271 controls [76]. Technological advances have provided the opportunity for studies with a higher number of genotyped ALS cohorts. The largest release of ALS genomic data was published in 2018 by Nicolas et al., and identified *KIF5A* as a novel ALS-associated gene; the study included a publicly-available large meta-analysis dataset of 10,031,630 imputed SNPs of 20,806 ALS and 59,804 controls as well as providing controlled access to “raw” genomic data including SNP-arrays of 12,188 cases and 3,292 controls [12,17]. Despite that hundreds of ALS-associated variants have been recorded in public databases such as the GWAS Catalog [10], these associations show very little reproducibility across different studies and have not been able to explain a large percentage of ALS heritability [3,36]; a phenomenon which is generally known as the “missing heritability” paradox [77]. It has been proposed that SNPs contribute ∼8.5% of the overall heritability of ALS, although it should be noted that such estimates consider only linear single-marker effects of SNPs [36,77]. Here we outline some general GWAS limitations in the context of ALS, as well as potential reasons why standard GWAS phenotype-genotype analysis is unlikely to fully explain the genetic architecture of ALS.

A first general challenge in large scale genomic analyses is to ensure a high quality of the genotype data, so that the downstream results of the experimental design reflect true biology and not artifacts. Therefore, the collected genomic data first need to pass a comprehensive Quality Control (QC) pipeline including multiple sample and variant QC steps [78,79,80,81]. One challenge is that each dataset has its own specific features, thus there are not fixed thresholds for each quality-control step. For this reason, each study needs to follow a data-driven approach, taking into consideration the distribution of each data metric. However, there are some good practices in QC that may be generally applicable to most studies [78,81]. For example, it is typical to follow a procedure first filtering out low quality samples then removing poor quality markers, the order of this ensuring that as many genetic markers as possible are kept in the final dataset. However, overly strict thresholds can lead to the loss of a substantial proportion of samples, reducing study power. Another challenge is to ensure homogeneity of the collected samples in terms of ancestry. This QC step is carried out by analysing the population structure to remove ethnic outliers, and by accounting for confounding factors in later stages of the analysis, such as a potential inner population sub-structure, usually using the first few Principal Components, after performing a Principal Component Analysis on the homogeneous sample cohort. Also, it is very important to check for duplicated samples and, in non-family GWAS analyses, ensure that all samples are unrelated so that specific genotypes are not over-represented (and thereby contributing a bias to the subsequent analysis). Identity-by-descent (IBD) is a metric that corrects for such bias and takes into account the number of variants that a pair of individuals share.

GWAS is a single marker analysis treating each variant association as an independent event that contributes to the phenotype. Due to this, it is a standard practice for results to be corrected under the strict multiple testing threshold (*p* < 5 × 10${}^{-8}$) of the Bonferroni correction in order to control for false positive discoveries (Family-wise type I errors). This threshold derives from the hypothesis of 1,000,000 independent markers being tested under a significance level of 5%. Particularly in low sample size studies this correction can result in a loss of power of the analysis, which may then fail to capture a portion of potential risk variants that do not pass the significance threshold (Family-wise type II errors) [15,82].

Univariate analyses such as GWAS that test trait association for one locus at a time are not able to capture multilocus interactions-a phenomenon called epistasis-and the interaction of the environment with the genome; events that could potentially account for the missing heritability of ALS and explain the disease pathology [83,84]. The term *epistasis* was introduced in genetics over a century ago by Bateson et al. [85], and genetic and evolutionary biology studies have highlighted the importance of gene-gene interactions not only in the genetic architecture of an organism but also in evolution [77,86,87]. Epistasis represents non-additive events in the genome including interactions among two or more loci that have an effect on the phenotype [88]. Several studies have highlighted the role of epistasis in pathology, showing that SNP interactions provide a stronger association to the disease than the participating SNPs do individually [77,84,89,90]. To understand pathology in a complex disease such as ALS, it may be necessary to identify complex genetic interactions, including epistatic interations [77,87]. Nevertheless, the study of multilocus interactions poses a number of challenges, in particular the need for a high computational power as the number of tested interactions is extremely high even in pairwise combinations. As such, multivariate computational approaches and appropriate machine learning methods may be able to capture the potentially complex relationships among risk variants in ALS [77,90,91].

GWAS is more successfully employed under a “common disease-common variant” hypothesis, being of particular use in common diseases such as schizophrenia which are driven by many risk alleles each with high frequency [92]. In contrast, ALS is a heterogeneous disease likely comprised of multiple strata each resulting from combinations of different rare mutations and other factors. As a result, stratum-specific mutations may each have very small effects that are diluted and thus not captured by GWAS [3,36]. The majority of GWAS analyses have used SNP-arrays as they have until recently had a lower experimental cost in comparison to sequencing of the exome or the whole genome. SNP-array analyses can typically capture the effect of only common variants to the phenotype whereas sequencing analyses identify both common and rare variants. In most SNP-array GWAS studies, variants with Minor Allele Frequency (MAF) of <1–5% are removed from subsequent analysis as they are generally more difficult to genotype and therefore are considered potential false positives [15,78]. Nevertheless, whole genome sequencing, custom designed exome sequencing arrays, rare variant burden analyses and imputation approaches using large reference panels (such as the Haplotype Reference Consortium, which contains 64,976 haplotypes), face this challenge by recovering both rare (up to 0.1% MAF) and common variants that SNP-array platforms do not usually contain [15,93,94,95]. However, there is still a proportion of low frequency minor allele effects on the phenotype that cannot yet be detected by GWAS approaches and that could also potentially explain some of the missing heritability in ALS [3,36].

Lastly, another common GWAS challenge in complex diseases is the difficulty to distinguish causal variants from other non-disease-associated variants that are in high linkage disequilibrium [15]. Linkage disequilibrium describes the phenomenon where an allele of a variant is inherited together with the alleles of other variants [9]. These alleles of other variants are highly correlated and will have very similar GWAS signals with the truly causal SNP. The majority of disease-related variants are located in cis-regulatory regions of the genome [96], and given our limited knowledge of non-coding genomic loci, it is even more challenging for those to discern causal SNPs from the noise. Our difficulty to identify the causal variants in complex diseases among a pool of statistically significant associated variants adds to the challenge of identifying molecular processes that could have a significant impact on the disease.

**Figure 1:**
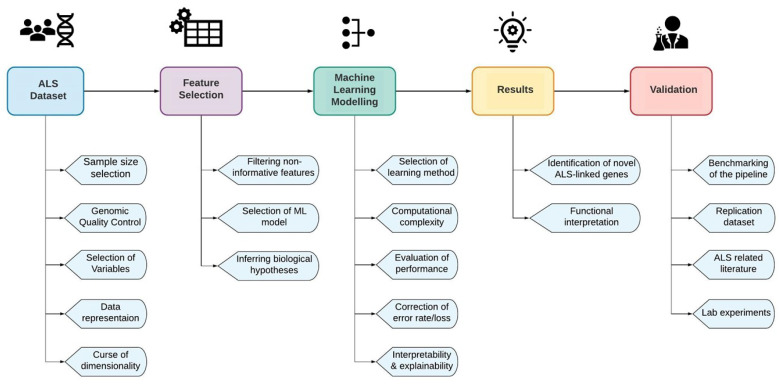
The main challenges of an ALS machine learning experimental design.

Advanced machine learning prediction models trained in ALS genomic data could overcome the aforementioned challenges, moving towards better insights into disease causality and ultimately to a personalized understanding of ALS [15,97]. In Figure 1, we describe the basic steps of an ALS machine learning experimental design in order to discover ALS-associated novel loci or combinations of loci, as well as the main challenges of each step. Each of the main challenges is addressed in successive chapters of the review, as we describe and compare the experimental design of the collected gene prioritization studies. Some of the challenges in Figure 1 have already been mentioned, such as the need for a *large sample size* that could increase the power of the study, a *comprehensive quality control pipeline* to assure high quality genomic data, as well as the *curse of dimensionality* which is a very common problem in genomic studies that include an extremely high number of features and especially in studies that focus on multilocus interactions.

## 3. Facing the Challenges

In this chapter, we outline gene prioritization approaches by which published research studies have employed machine learning methods in order to identify and rank novel ALS-linked genes, SNPs and multilocus interactions. First, a short introduction is made to some basic machine learning concepts that will be useful in later discussion of the machine learning approaches that have been used. Then, details are provided about the data representation, feature curation, and selection methods that each study chose for their experimental design and, finally, the machine learning methods and the overall experimental designs of each study are compared, as well as considering their results.

### 3.1. A Brief Overview of Machine Learning Concepts

Based on the task and the type of learning there are three main machine-learning categories: supervised, unsupervised and semi-supervised algorithms. Supervised learning methods aim to make predictions on unknown instances (e.g., a sample or a gene) based on known labels (e.g., ALS/non-ALS) [98]. For instance, classification is a supervised machine learning approach, which trains a classifier using labeled data e.g., samples/genes including a case/control label (training set) and predicts the class of an unknown sample or gene (testing set) based on specific rules and patterns that the classifier learned during training and testing.

In classification, it has always been a challenge to identify the input features that are most informative and maximally affect the prediction. This domain of research is described as *feature selection* research [99] and has been traditionally connected to statistical methods. More recently, with the advent of deep neural networks, *explainability* and *interpretability* of machine learning suggestions, predictions and decisions has become an even more acute problem. This is due to the complex nature of the network itself, which does not clearly illustrate the connection between input (features) and output (prediction) in a humanly understandable manner. Thus, a number of recent studies aim for *explainability* in deep models [100], including visualized explanations [101].

On the other hand, unsupervised learning is performed on unlabelled data, with an ultimate purpose to identify interesting patterns or novel sub-groupings of the data. Clustering research offers a well-established set of unsupervised algorithms which identify patterns in a group of instances (e.g., Genes, SNPs, ALS patients), most commonly based on notions of distance (or similarity) between instances. Distance in such cases can be measured with metrics such as Euclidean distance or the Pearson Correlation Coefficient [74].

Lastly, in semi-supervised learning the prediction is carried out in positive and unlabelled data. Semi-supervised learning is applied when information is available only on instances of a single class (usually called “positive” instances, e.g., already known-ALS genes) but there is not sufficient information to label the rest of the instances as “negative”. Semi-supervised learning methods can be quite challenging when the aim is to predict novel disease-associated instances since the classifier is trained treating potentially novel instances as “negative”. Semi-supervised learning methods are particularly common in gene prioritization studies.

An *instance* in a machine learning task is an entity that the classifier is trained to predict, for example a gene in a gene prioritization algorithm or an ALS sample in an ALS/non-ALS patient classification task. An instance is described by a number of *features* (e.g., gene functional annotations, SNP genotypes etc.), typically represented as a vector, termed *feature vector*. All X instances (e.g., samples or genes/SNPs) need to have the same number of Z features (e.g., genetic mutations or functional annotations), leading to a two-dimensional matrix K that has a size of K = X * Z. Each instance has a specific location on a Z-dimensional space (where Z is the number of features) and each feature has, accordingly, a specific location in X-dimensional space (where X is the number of instances).

There are a wide variety of metrics that can be used to evaluate the performance of a machine learning model, the choice of which depends on the nature of the task. The most popular metrics include Accuracy, Precision, Recall, F1-score, Specificity and ROC (Receiver Operating Characteristic) curves. Accuracy represents the fraction of true positive and true negative predictions out of the total number of the model predictions. Precision expresses how many positive predictions of the model were truly positive. Recall (True Positive Rate) explains the percentage of the missed true positive predictions. Recall and Precision are both very important metrics that need to be taken into account for the evaluation of a model’s performance. F1-score calculates the harmonic mean of those two metrics, hence the higher the F1-score is, the better the model performed. Specificity expresses the fraction of the negative predicted instances that were actually negative. The ROC curve is a plot of True Positive Rate against False Positive Rate expressed as 1-Specificity. The Area Under the Curve (AUC) of ROC is employed to calculate how well the model performed - the closer the AUC is to 1, the better the model performed.

### 3.2. Recent Feature Preparation and Selection Approaches in ALS Genomic Studies

Modern analytical genomic platforms and imputation methods have led to the profiling of samples containing up to tens of millions of genetic markers. This vast amount of genomic information makes machine learning modelling more complicated and demanding, as it is likely that only a small minority, if any, of markers are truly causal and associated to the disease. Thus, a first challenge in the genomic machine learning experimental design is to deal with the *curse of dimensionality* using appropriate feature selection methods [102]. Below we describe and group the feature selection and dataset curation approaches that have been used by each study, also summarized in Table 1.

The use of feature selection methods are a common strategy to reduce the high number of initial features for machine learning models [102]. As described in Table 2, four of the seven studies used a machine learning model as a feature selection method, in some cases using specific hypotheses or along with a combination of other strategies that will be described later in this sub-chapter. Mantis-ml is one example of a multi-step gene prioritisation framework which extracts a heterogeneous set of 1249 gene-annotation features mined from a large collection of databases in order to discover and rank novel disease-related genes [103]. Each instance in this model consists of 18,626 coding genes which are labelled as positive (seeds) or unlabelled, depending on known association to the disease, based on information retrieved from the Human Phenotype Ontology (HPO) [109]. A number of pre-processing steps were applied that included filtering of highly correlated pairs of features, removing features with missing data, and imputing certain features with a low missing rate. Some exploratory analyses are then performed (i.e., heat maps, variable distributions, etc.) in the original feature space and then three dimensionality reduction methods are automatically applied: principal component analysis (PCA), t-distributed stochastic neighboring embedding (t-SNE) [110], and uniform manifold approximation and projection (UMAP) [111], in order to identify any interesting pattern(s) and linear/non-linear relationships among the features. The gene pool is randomly split into K balanced datasets containing both positive and unlabelled genes. Mantis-ml can use the Boruta feature selection algorithm which labels the features (given a decision threshold) as “confirmed”/”tentative”/”rejected” by assessing the contribution of each feature to the prediction [103], although the entire feature space is used by default.

Several recent studies have focused on uncovering gene-gene interactions (epistasis) that could potentially explain some part of the missing heritability of complex genetic traits such as ALS. However, modern genomic studies that aim to model epistatic events face considerable challenges, demanding large computational and statistical power for the analysis of a large number of combinations among millions of genotyped loci (even when considering only pairwise interactions). Two of the studies -Greene et al. and Kim et al.- included epistatic events in their feature selection approach [91,107]. Both used the wrapper method Multifactor Dimensionality Deduction (MDR)-a non-parametric, model-free approach which reduces the feature space of multilocus combinations by creating new single variables pooled from multiple SNP genotypes [87,112]- and then estimates statistically significant ALS-risk pairwise interactions of SNPs. Both studies used the same datasets and pre-processing steps to identify pairwise SNP interactions that are significantly associated with ALS. The datasets included two sporadic ALS cohorts along with healthy controls containing 276 cases versus 271 controls in the detection dataset and 211 cases versus 211 controls in the replication dataset [76,106]. SNPs were filtered using a 0.2 Minor Allele Frequency cut-off and a less than 90% call rate. Lastly, Greene et al. considered only independent SNPs in further analysis, leading to a dataset of 210,382 SNPs.

Another strategy to reduce feature space is to include only regulatory elements as features for the subsequent machine learning experiments. Two of the studies focus only on the effect of noncoding regulatory elements in ALS pathology. It has been shown that disease-related variants are mostly located in cis-regulatory elements of the genome marked by DNase I hypersensitive sites (DHSs)-zones of the genome that have been associated with elevated levels of transcriptional activity [96]. In 2020, Yousefian et al. investigated the effect of noncoding variants in ALS [104]. Firstly, they applied a *p*-value threshold (*p* < 5 × 10${}^{-4}$) on SNPs from a previously published large ALS meta-analysis GWAS dataset. The authors constructed association blocks by identifying lead SNPs having strong ALS GWAS *p*-value associations and being at least 1 Mb apart from each other, then selecting also the top 30 ALS-associated SNPs located upstream and downstream of each lead SNP; leading ultimately to 274 association blocks [104]. They enriched their selected association blocks with functional and epigenetic information including DHS profile data, histone modifications, functional gene-sets from the KEGG database and TF binding sites collected from TRANSFAC and JASPAR databases [104,113,114,115]. After the functional enrichment of the features, they constructed a binary feature matrix representing whether a SNP within an association block was associated or not with a particular functional feature [104]. The second ALS study that included only noncoding regulatory elements was published in 2019 by Yin et al. who proposed Promoter-CNN as a feature selection method; a convolutional neural network model (comprised of 2 convolutional layers and two deep layers) that reduces the initial feature space by selecting only the top 8 highest performing promoter regions among variants located on chromosomes 7, 9, 17 and 22 [90]. They assessed the performance of Promoter-CNN using a 9-fold cross validation. Each promoter region of each individual was represented by a window of 64 genomic features having a value of 0,1,2 to represent the genotype at each of these 64 loci, utilizing the genomic structure of the regions [90]. The aforementioned studies employed the genomic structure in order to build the association blocks and the promoter regions (see Table 1).

A popular strategy that has been used to select the initial data and then further reduce the feature and instance space is ALS-associated knowledge (see Table 1). The most relevant study that falls into this category is Bean et al. which modelled only previously known ALS-linked gene lists, mining information from the literature and from disease databases, as well as including a manually curated set of ALS-associated genes [105]. In order to reduce the feature space they performed an enrichment test on all features and, for the predictive model, kept only those features that are significantly enriched in the mechanism(s) of the disease [116]. Another example is Yin et al. which before applying their Promoter-CNN model as a feature selection method, they first limited their feature space by studying only non-additive phenomena of multiple promoters located on four specific chromosomes (7, 9, 17 and 22), those chromosomes have being selected based on the amounts of missing heritability that have been previously identified in ALS [36,90]. Bean et al., Kim et al., Yousefian et al. and Sha et al., implement multi- step algorithms in which one of the initial steps reduces the feature space by keeping only the highest performing genes/SNPs, by assessing the enrichment of genes in ALS [105] or by applying a specific threshold of single-marker association analysis to ALS [91,104,108].

### 3.3. Experimental Design and Results of the ALS Gene Prioritization Approaches

In this section we will focus on the approach and rationale of the collected studies that aim to understand the pathology of ALS using machine learning and probabilistic models on genomic data. We also briefly consider the main findings of each study. Although the majority of the collected studies aim to answer more than one research question, in this section we group studies by methodology, based on common features of their experimental design that we considered to be the main focus in each study. In order to avoid confusion, in Table 2 we describe the main biological focus of each study. We investigate the reproducibility of the results of each analysis, comparing it with related literature as well as mentioning putative novel ALS discoveries in the Discussion section.

The identified studies (see Table 1) all fall under the gene prioritization umbrella category. In Table 3, we provide brief information about the machine learning models that have been used in these studies (after feature selection and filtering approaches have been applied, as discussed in the previous section, see Table 1), as well as details about the assessment and the performance of the models.

Four of the studies included epistatic events in their experimental design, testing the hypothesis that multilocus interactions have an effect on ALS susceptibility (see Table 2). Three studies that fall into this category, all use the same ALS detection dataset in order to discover pairwise SNP interactions [91,107,108]. Greene et al. and Kim et al. use multifactor dimensionality reduction (MDR); a wrapper method which performs both a feature selection and classification to predict pairwise combinations of SNPs as high-risk and low-risk for ALS [91,107]. As proposed by [112], MDR reduces multilocus dimensions into a one-dimensional multilocus variable, the prediction performance of which is evaluated in classification tasks (ALS versus healthy controls) by cross validation and permutation tests. Out of pairwise combinations among 210,382 SNPs, Greene et al. reported the pair of SNPs rs4363506 and rs6014848 to have the highest accuracy (Acc: 0.6551) and a *p*-value of 0.048 after permutation testing. Their replication dataset showed a lower accuracy (Acc: 0.5821) but with a higher statistical significance (*p*-value < 0.021) [107]. Kim et al. chose the best performing MDR model for each SNP, then they mapped SNPs to genes (including neighboring regulatory elements) and investigated their enrichment using Gene Ontology functional terms [91]. Unfortunately, they did not report any specific high performing pairs of SNPs in terms of MDR accuracy, nor specific genes. The statistical significance of each MDR model was estimated using permutation, with the highest enriched Gene Ontology gene-sets being “Regulation of Cellular Component Organization and Biogenesis” (*p*-values: 0.010 and 0.014), and “Actin Cytoskeleton” (*p*-values: 0.040 and 0.046). The *p*-values for each gene-set refer to the detection and replication dataset respectively, after multiple testing. The third study is proposed by Sha et al. implementing a two-stage probabilistic algorithm that attempts to predict two-locus combinations that are associated to ALS using eight epistatic and nine multiplicative two-locus predicting models. The first step was a single-marker association test using the $x^{2}$ test statistic, keeping only the 1000 SNPs having the strongest *p*-values. The associations of all the two-locus combinations of those 1000 variants were then tested using the above-mentioned seventeen models. Three SNPs were discovered participating to two two-locus combinations: rs4363506 with rs3733242 (*p*-value = 0.032) and rs4363506 with rs16984239 (*p*-value = 0.042). Reported *p*-values were adjusted for multiple testing correction using permutation. They also performed Multifactor dimensionality reduction and Combinatorial Searching Method to identify high performing two-locus interactions, but none of the results reached the significance threshold (see Table 3). Single locus analysis was not able to capture the three SNPs that participate to the bi-locus interaction.

A far more complex machine learning model for ALS patient classification, studying non-additive interactions of multilocus promoters among four chromosomes, was published in 2019 by Yin et al. [90]. Building upon previous knowledge that the majority of the disease-associated variants in GWAS are cis-regulatory elements, this study showed that using only the highest performing promoter regions of 4 chromosomes as features provides enough information for successful classification of the ALS genomic profile versus healthy controls [90]. Specifically, they applied a prediction model for putative ALS-related genotypes located in promoter regions, using a case-control genomic dataset containing 4,511 cases and 7,397 controls from the Dutch cohort of ProjectMinE. A two-level pipeline was constructed in which, as a first step, deep neural networks select the eight highest performing promoter regions of chromosomes 7, 9, 17 and 22 having the highest accuracy in ALS status prediction (Promoter-CNN model) and then the selected promoter regions of each individual are combined for a final classification task (ALS-Net model). A 9-fold cross validation was used to train both models. The authors compared their ALS-Net deep learning model to other classification models using both the pre-selected promoter regions combined from all four chromosomes by Promoter-CNN models, and markers from individual chromosomes. The compared classification models included a logistic regression Polygenic Risk Score (PRS) approach [117], Support Vector Machines (SVM) [118], Random Forest [119] and AdaBoost [120,121]. The two-tier deep learning model showed very promising results identifying both already reported associated ALS genes and new putative ALS markers. The results showed that ALS-Net combined with Promoter-CNN pre-selected promoter regions produced better performance (Acc: 0.769, F1-score: 0.797) than the logistic regression Polygenic Risk Score (PRS) approach (Acc: 0.739, F1-score: 0.728) [117], Support Vector Machines (SVM) (Acc: 0.725, F1-score: 0.694) [118], Random Forest (Acc: 0.596, F1-score: 0.381) [119], or AdaBoost (Acc: 0.661, F1-score: 0.625) [120,121]. They highlight that their Promoter-CNN model is a successful feature selection method keeping only the highest performing promoters from each chromosome individually, advancing the performance of the subsequently tested classifiers [90]. The findings indicate that combining genomic information from all four chromosomes improves the performance for the majority of the models, supporting further the hypothesis that non-additive events take place in ALS pathology.

Another recent study that builds upon the hypothesis that cis-regulatory noncoding variants can have an important effect on ALS pathology, applies a functional SNP prioritization framework using convolutional neural networks (CNN) to make ALS rare noncoding risk-variant predictions [104]. The authors build upon a previously published deep learning CNN-based model that used functional features in order to predict causal regulatory elements in complex diseases [122]. They tested their proposed method on a large GWAS meta-analysis cohort including 8,697,640 SNP *p*-values for 14,791 ALS patients and 26,898 healthy controls [12]. The functional SNP prioritization framework followed a multi-step procedure starting from (a) collecting the upstream and downstream flanking regions of the 274 highest GWAS ALS-associated variants, then (b) functionally annotating the variants (including DNase I hypersensitive sites (DHSs), histone modifications, target gene functions, and transcription factor binding sites (TFBS)) and finally (c) training the CNN model on these association blocks with uncertain class labels using chromosomes 1–10 as a training set, chromosomes 11–14 as a testing test and finally 15–22 as a validation set [104]. The CNN model used two convolutional layers; the first layer measured how well each individual SNP matched the pattern of 50 functional features using a rectified linear unit (ReLU), and the second level output prediction scores for each SNP with a 0–1 range, with values close to one indicating that there are common regulatory patterns embedded for a particular SNP. The CNN model shows a high predictive performance (AUC = 0.96 and F1 = 0.83). A random forest classification for the ALS cell-type specificity showed that a high portion of their ALS selected features have neuronal cell-type specificity within Trancriptional Factor binding sites. The proposed framework highlights two potentially functional ALS-risk variants rs2370964 (chromosome 3, located in enhancer site of *CX3CR1*) and rs3093720 (chromosome 17, intron variant in *TNFAIP1*). An eQTL analysis was performed to investigate the effect of these two variants on the expression of other genes. The analysis showed that the two noncoding variants may impact ALS risk by affecting the expression levels of *CX3CR1* and *TNFAIP1*. The *CX3CR1* gene deletion has been associated with microglia neurotoxicity and neuron loss in transgenic ALS mice [123,124]. TNFAIP1 is an apoptotic protein which has been associated to neurotoxicity [125]. The rs2370964 variant also affects the *CTCF* and *NFAT* binding sites [104]. Mutations in *CTCF* gene has also been related to microglial dysfunction, among other effects [126]. NFAT is a transcription factor that is involved with the regulation of pro-inflammatory responses in cultured murine microglia [127]. The rs3093720 SNP affects the *NR3C1* binding site, a gene which has been associated with neurodegeneration and multiple sclerosis [128].

The accumulation of large amounts of ALS multi-omic data, functional annotations, and tissue-specific information, has provided a great opportunity and challenge for researchers to combine these in ways that could potentially lead to stronger machine learning models. Two collected studies combine known ALS genes and ALS-related information mined from a variety of databases to predict novel disease-specific genes and rank them. The first one is, Mantis-ml, a recently published multi-step disease agnostic gene prioritisation pipeline which employs known disease-associated genes to predict scores for putative novel genes based on feature pattern similarity [103]. As mentioned in Section 3.2, the disease-related gene information is extracted from multiple databases and resources, including tissue- and disease- specific data [103]. Depending on the users’ disease-related queries, the pipeline follows automatic feature selection and pre-processing as well as an exploratory data analysis on disease-related features. A repeated stochastic semi-supervised model is used to iteratively predict disease-related probabilities for each gene, and then rank each gene based on the mean prediction probability of all iterations. The starting point for the modelling is the labelling of an entire coding gene pool (18,626 genes) based on disease relevance retrieved from the Human Phenotype Ontology (HPO), with positive and unlabelled genes then being split into random balanced datasets [109]. They evaluate the performance of their classifier using a stratified 10-fold random split in every balanced dataset, which is followed by testing using the out-of-bag k-fold method. The model generates gene prediction probabilities belonging to the respective testing set of each k-prediction cycle. Lastly, the model calculates an aggregated prediction probability combined from all iteration cycles. The classification performance of Mantis-ml was assessed using seven different supervised models (gradient boosting, random forest, extra trees, extreme gradient boosting, support vector classifier, deep neural networks, and a stacking classifier) with 10 stochastic iterations and 10-fold cross validation in three diseases. All models showed similar performance (AUC: 0.83–0.85), with Extra Trees having the highest mean Area Under Curve for ALS (AUC = 0.814) (see Table 3). For ALS, 77 positively labelled genes were selected, having an average AUC of 0.814 (combined from 7 classifiers). Among the top 50 genes there were two already known ALS associated genes, *FUS* and *L1CAM*. Specifically, “MGI mouse knockout feature” was ranked as the top feature for ALS, including human orthologue mouse genes that have been associated with survival and developmental pathways. Unlabelled genes (i.e., genes not annotated to ALS in the HPO) were also identified as being predictive of ALS. Some of the top novel predicted genes among 5 out of 6 classifiers are *SYNE1, ALDH5A1, ABCA1, DNMT3A, NF2, SZT2, ACADVL, MED12, TSC2, EP400, RYR2, VCL* and *BBS2*. *SYNE1* causes recessive ataxia and has been associated with motor neuron degeneration and ALS [129]; ALDH5A1 has been identified as significantly down-regulated protein in ALS murine models [130]; ABCA1 has been linked with damage of neuromuscular junctions and identified in significant clusters of altered frontal cortex genes in ALS samples [131,132]; a DNMT3A isoform has been identified in synapses and in mitochondria and has been associated with degeneration in motor neurons in ALS patients and abnormal expression levels in skeletal muscle and spinal cord of presymptomatic ALS mice [133,134].

Another study that combined genomic data with other types of data sources to increase the power of the machine learning gene prioritization method was that of Bean et al., integrating functional annotations, known ALS-gene associations, and protein-protein interactions [105]. Protein-protein interaction networks have been useful to decipher new disease mechanisms, as proteins that are encoded by disease-related genes are likely to interact with proteins that are implicated in similar pathologies [135]. These authors used a previously published knowledge graph-based completion model [116], which is trained combining protein-protein interactions data mined from Intact [136,137], known disease-gene lists from DisGeNet [138,139] and functional gene-sets from the Gene Ontology [140] in order to make predictions of novel ALS-linked genes [105] (see Table 1). The algorithm starts by building a knowledge graph containing known ALS data represented as nodes and their interrelationships as edges. The aim of the model is to predict the missing edges of the graph that could represent novel ALS genes, providing a predictive score to each. This is a similarity score deriving from the profile which is built by the trained knowledge graph model comparing the ALS-known genes to the rest of the genes. They train 5 models for 5 input sets of ALS-linked genes mined from the ALS Online Database (ALSoD), which is intended to host all known ALS- associated genetic variants [18], from the ClinVar database of curated clinical variants [141], from DisGeNet [138,139], and a manual curated ALS gene list generated by the authors [105] (see Table 1). For the training and testing of the model, 5-fold cross validation was used to estimate how well the model would have predicted known ALS genes. As seen in Table 3, all models-except the DisGeNet list- performed very well in each fold above the random baseline, with the manual list being on top. In total, the 5 models predicted 45, 176, 192, 327 and 575 novel ALS for the Manual list, DisGeNet, ClinVar, ALSoD and union ALS-known lists, respectively. All predicted novel genes of the manual list were also present in the other 4 lists. The authors also tested the functional enrichment of the predicted genes following an overrepresentation analysis using Gene Ontology terms, with all gene-sets having statistically significant enrichment to ALS-specific biological processes, like mitochondrial activity, endosome transport and vesicular trafficking, lipid metabolism and others. To validate the relevance of the predicted ALS genes, a gene-set and gene-level analysis was performed using MAGMA [142] on a large ALS meta-analysis GWAS dataset (European cohort, including 20,806 cases and 59,804 controls) [12], keeping only the variants that mapped to the putative ALS-genes. Only ClinVar predicted genes had statistically significant results (*p*-value = 0.038), followed by the Manual model which did not pass the Bonferonni correction but it was close with a *p*-value of 0.060.

## 4. Discussion

**Figure 2:**
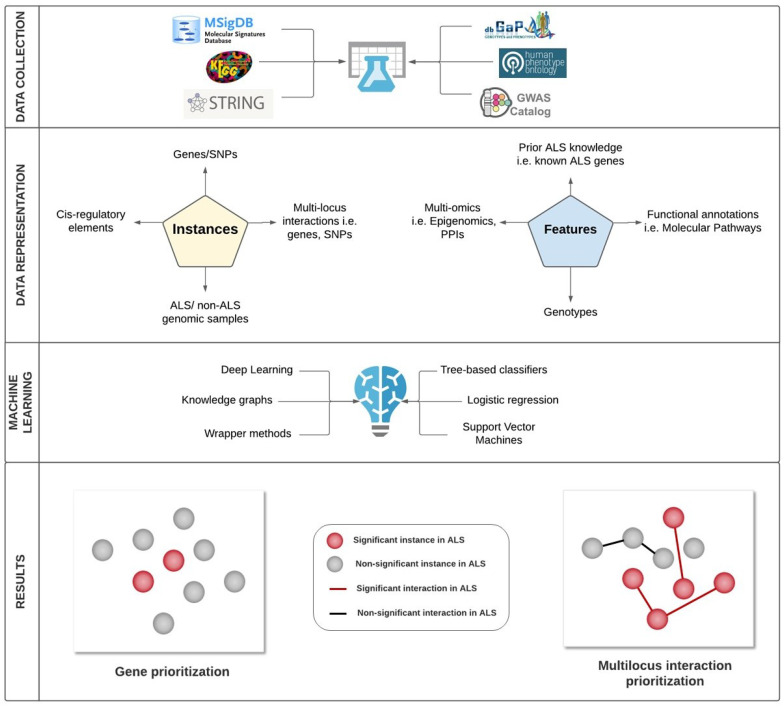
Some of the key decision making steps taken by machine learning approaches in ALS genomics, and examples of some possible choices at each step. These included data collection, data representation, selection of the Machine Learning algorithm for the classification task, and the types of result obtained by the model. The studies collected information from a variety of databases in order to mine, among other things, data on genotypes (e.g., dbGaP, GWAS Catalog, gnomAD), functional annotations (e.g., KEGG), and Protein-Protein Interactions (e.g., STRING). Depending on the purpose of the experimental design, the collected studies modelled genes, SNPs, cis-regulatory regions, multilocus interactions, and/or ALS/non-ALS patients. Each instance was described using features such as Genomic, Epigenomic, and Proteomic data, functional annotations, and/or prior ALS-related knowledge (e.g., ALS gene-sets). Various machine learning algorithms were selected for the classification tasks. Lastly, we visualize the distinction between the modelling results of gene prioritization and multilocus interaction prioritization studies. Gene prioritization studies aim to identify significant ALS associated instances (e.g., SNPs, genes and cis-regulatory regions), whereas multilocus interaction prioritization studies aim to discover significant interactions among multiple loci. Lastly, we note that an ALS versus non-ALS sample classification experiment can be used to prioritize genes if the interpretability of the chosen model permits the identification of informative genomic features.

Here we identified gene prioritization machine learning studies aimed at the understanding of genomic data in ALS, outlining the main challenges faced by such studies. We compared these studies in terms of their feature selection methods, experimental design, machine learning performance and their biological results. In Figure 2, we summarize some of the key decision making steps taken by machine learning approaches in ALS genomics, and examples of some possible choices at each step.

In gene prioritization studies, the initial number of potentially “novel” SNPs/genes or, more problematically, the potential novel multilocus combinations, can be so high as to make computational analysis unfeasible (see Figure 1). In this context, it was intriguing to group and compare the chosen dimensionality reduction approaches in each study. As seen in Table 1, most studies handled this problem quite differently. More than half used a machine learning method for feature selection, along with one or more biological hypotheses for additional filtering of the input variables. The most common approach of biological hypothesis filtering was to infer ALS-specific knowledge early in the experimental design. This is both an advantage and a potential disadvantage as it makes the results more likely to have biological relevance but at the same time risks introducing bias to later stages of the machine learning approach. An example of a feature selection method that fell into this category is an early SNP-filtering approach based on a specific threshold of ALS GWAS *p*-values. This approach succeeds in a straightforward way to reduce the feature space, but comes at the cost that potential GWAS false positives could be inferred and/or that true positives (not captured by GWAS) might be removed from further analysis. This may be especially problematic in the capturing of epistatic events, as traditional GWAS analysis is a linear single-marker analysis, so filtering based on single SNP-disease association *p*-values could risk losing putative significant multi-locus interactions in later stages of the analysis [143,144]. However, a large cohort size could increase the power of a standard GWAS analysis utilized as a feature selection method in a machine learning study.

Deep learning seems a promising machine learning method for ALS gene prioritization studies, as well as in ALS patient classification. Deep learning methods are known to perform well in regulatory genomic classification tasks, being able to incorporate information from the structure of the genomic data and capturing non-linear relationships and patterns of multilocus interactions [145,146]. This statement is further validated by comparing the performance of three collected ALS gene prioritization studies. More specifically, three recent studies that employ deep learning as at least one of their classification methods had very good predictive performance [90,103,104], as summarized in Table 2. Yin et al. showed that deep neural networks perform better than other methods in classifying ALS versus healthy controls, not only in terms of accuracy (0.769) and F1-score (0.797), but also with an excellent recall of 0.908, using a 9-fold cross-validation [90]. Moreover, all of the benchmarked classification methods showed improved performance when promoter selection Convolutional Neural Network models (Promoter-CNN) where incorporated as an extra feature selection stage with the classification models. Yousefian et al. constructed a semi-supervised Convolutional Neural Network model in order to predict ALS-associated non-coding variants using epigenetic features. The model showed an excellent performance achieving an AUC of 0.96 and F1-score of 0.83 [104]. This study did not include benchmarking against other machine learning models, and interestingly the association blocks of non-coding variants that were constructed for training, testing, and validation sets of the model were separated into chromosome numbers 1–10, 11–14 and 15–22, respectively, rather than the cross validation methods typically employed by other studies. Lastly, Vitsios et al. benchmarked their proposed multi-step non-disease specific gene prioritization pipeline assessing the performance of seven classifiers using 10-fold cross validation in three diseases. In ALS, all classifiers achieved very similar performance with an average AUC ranging from 0.767 to 0.814, with Deep Neural Networks achieving an average AUC of 0.774 and Extra Trees classifier being at the top [103]. Even though, the majority of the ALS collected studies show that deep learning yields very good classification results, one very popular challenge in such learning algorithms is the lack of explainability in the model’s results, identifying which features are the most informative to the classification task.

In terms of reproducibility, comparing the highest ranked genes and SNPs, as well as the reported statistically significant functional pathways, it is noteworthy that the sALS-associated variant rs4363506 (initially identified by Schymick et al. with an empirical *p*-value = $10^{-6}$ [76]) was found by both Greene et al. and Sha et al. to have statistically significant pairwise interactions with other variants. Specifically, Greene et al. reported rs4363506 to interact with rs6014848 (Acc of 0.6551 and a *p*-value < 0.048 in the detection dataset, and Acc of 0.5821 and *p*-value < 0.021 in the replication dataset) and Sha et al. found that rs4363506 participated to two separate two-locus interactions: one with rs3733242 (*p*-value = 0.032) and another with rs16984239 (*p*-value = 0.042) [107]. These were the only significant pairwise combinations identified by either study. The replicated SNP rs4363506 is an intergenic variant (chr10:127476239, GRCh38.p12) located between *DOCK1* dedicator of cytokinesis 1 and *NPS* neuropeptide S [147]. *DOCK1* is implicated in neural growth and is a member of the KEGG pathway term “Regulation of actin cytoskeleton” and *NPS* in “positive regulation of synaptic transmission, glutamatergic” (GO:0051966) and “regulation of synaptic transmission, GABAergic” (GO:0032228), among others, all processes that are linked to ALS (see Section 1.1) [113,148]. This is consistent with other work implicating the actin cytoskeleton in ALS, including the finding of the third study, Kim et al.-investigating the functional enrichment of pairwise interactions in sporadic ALS-of statistical significance of the Gene Ontology term “Actin Cytoskeleton” (*p*-value = 0.040). However, it should be kept in mind that all 3 of these studies are analyses of the same primary dataset.

There could be several reasons for the disparity in the results of Greene and Sha et al. (i.e., the identification of different interaction partner SNPs to rs4363506). One reason could be that different quality control methods and thresholds are applied to the genomic datasets. In addition, Greene et al. investigated the pairwise combinations of 210,382 uncorrelated SNPs (keeping only independent SNPs in terms of linkage disequilibrium) using their proposed MDRGPU model, whereas Sha et al. tested the pairwise combinations of the top 1000 SNPs with a significant ALS-association *p*-value, before testing for significant interactions using a series of different two-locus probabilistic models. The significant results of these studies also differed in the design of the predictive model used, with Greene et al. using Multifactor Dimensionality Reduction (MDR)-a non-parametric method combining feature engineering and then classification which does not make any assumption about the underlying genetic mechanisms-and Sha et al. using two-locus probabilistic parametric models which tested specific hypotheses about the type of the genotype interaction, taking into account the order of the genotypes in terms of penetrance of high-risk variants. Asides from the two-locus probabilistic models, Sha et al. did in fact also test (separately) an MDR and a Combinatorial Searching Method-unlike Greene et al., these analyses did not identify any significant pairwise combinations but, interestingly, SNP rs4363506 was present in a pairwise combination in both models that almost passed the threshold of statistical significance. Lastly, Kim et al. followed a multi-level approach starting from SNP pairwise interaction feature selection, moving to testing genes associations and gene-set functional associations to ALS. Unfortunately, they did not report any results about the MDR predicted pairwise interactions, hence we cannot make a direct comparison with the other two studies.

From the comparison of the most statistically significant genes and SNPs predicted from the optimally performing machine learning models of Mantis-ml [103], the knowledge graph completion model [105], the Promoter-CNN model [90], and the non-coding variant CNN model [104], we note several points. First, we compared the top 50 ALS genes predicted from the Mantis-ml model using Extra Trees-the highest performing classifier of the ALS data [103]-with the top 45 performing genes predicted by the knowledge graph-based machine learning approach using the highest performing model trained on the manually curated ALS-linked list [105]. *SLC1A2* (solute carrier family 1 member 2) was the only gene that was predicted by both approaches. SLC1A2 protein is the dominant transporter that clears the extracellular neurotransmitter glutamate in the synapses, expressed by astrocytes [149]. The down-regulation of *SLC1A2* has previously been associated with excitotoxicity leading to motor neuron degeneration and therefore contributing to ALS pathology, as described in the introduction (see Section 1.1) [47,48,150]. The Mantis-ml ALS model top predicted genes also had an overlap with the genes associated with the top performing promoters predicted by the Promoter-CNN model [90], but only in terms of shared protein families: within the top 8 promoter regions that Promoter-CNN selected for chromosomes 7, 9, 17, and 22, there were two promoter regions that were associated with genes *LAMB4* (laminin subunit beta 4) and *TRIM16* (tripartite motif containing 16); while among Mantis-ml highest predicted ALS genes were *LAMB3* (laminin subunit beta 3) and *TRIM28* (tripartite motif containing 28). The Laminin family contains heterotrimeric glycoproteins of the extracellular matrix that are associated with processes such as adhesion, survival, neuronal development and proliferation [151]. *LAMB4* is implicated in tissue development and cell migration, and has been associated with different types of cancer [140,152]. Interestingly, a laminin-4 isoform is expressed in neuromuscular junctions and has been associated with muscular dystrophy [151]. A recent study of *LAMB3* upregulation implicates this gene in cell apoptotic, proliferating and metastatic events in patients that suffer from pancreatic cancer [153]. *TRIM16* has been associated with autophagy, degradation of protein aggregates and ubiquitination of misfolded proteins; pathways that have been previously associated with ALS (see Section 1.1) [154]. Finally, *TRIM28* encodes a co-repressor protein which is expressed in the human brain and is a major regulator of transposable elements [155]. Elevated transcription of transposable elements has been linked with neurological disorders, including ALS, as well as binding to the ALS-associated RNA-processing protein TDP-43 [156].

It is noteworthy that the vast majority of the overlapping genes that we identified among all the collected studies are implicated in previously known ALS-associated functional pathways (as we outlined in Section 1.1) as well as the majority of the highest predicted novel genes (as described in Section 3.3). Nevertheless, we did not observe any further overlap among the other studies in terms of ALS-predicted genes. Limited reproducibility among the four studies could be due to multiple factors that derive from a number of differences in their experimental design and the focus of each study (see Table 1, Table 2 and Table 3). As summarised in Table 1, these four studies all use different instances, features and feature selection methods. As far as the machine learning models are concerned, the best performing machine learning models which were utilized for comparison were different. However, the low reproducibility could also derive from a more general challenge of gene prioritization studies, which concerns the difficulty of identifying the truly causal genes out of a usually large pool of novel predicted genes that pass a chosen significance threshold [157]. The difficulty of reproducibility is also emphasized in Bean et al. where 5 known ALS-linked gene lists mined from different databases, and one which was manually curated, were used, with each yielding very different results even with the same model, highlighting that the methodology and results of each study should be compared with caution [105]. Lastly, we need to mention that due to the high number of potential novel genes among all the benchmarked models of each study, we only compared the top genes/SNPs predicted by the highest performing model in each case. Hence, due to this limitation, we acknowledge the possibility of a larger existing overlap of the top genes predicted by the rest of the well performing models that were benchmarked among the studies. These challenges, makes the identification of the ALS implicated functional pathways even harder as well as the task of investigating reproducibility in different studies (see Figure 1) [157,158].

We also note that several studies incorporated prior ALS biological knowledge and functional annotations into the general experimental design (see Table 2), and the trained models showed a very good predictive performance (see Table 3). Specifically, as seen in Table 1 all of the considered studies except Greene et al. used an ALS-linked knowledge feature selection method to reduce their feature space as well as the number of instances (i.e., genes and SNPs). Moreover, Bean et al., Vitsios et al. and Yousefian et al. include ALS- specific and generic biological knowledge into their initial feature space, such as tissue/disease-specific features and known ALS disease-gene associations, as well as gene functional annotations, epigenetic features and protein-protein interactions (see Table 1). The most characteristic study that falls into this category is the one of Bean et al., where the instances were only ALS-linked lists from various resources, and the model was an ALS knowledge-based graph which uses the neighborhood of genes and enrichment tests to define significance of association, trained using PPIs, ALS-gene associations and functional annotations, showing the highest performance using a manually curated ALS-linked gene list.

Multilocus interactions may have a significant role in ALS and should be considered in future ALS genomic studies [90,91,107]. Each of the studies that investigated multilocus interactions has highlighted that the statistically significant variants could not be replicated in single-locus analyses [90,91,107,108]. Yin et al. carried out the most complex study in this category, using a large ALS cohort (see Table 1) followed by a thorough quality control -this was the first ALS multilocus study to investigate complex non-additive events in such a large scale of input variables. The results provided further support for the involvement of non-additive genetic interactions in ALS, showing that combining the genomic structure from multiple cis-regulatory elements (in this case promoters located in different chromosomes) yields very promising results in ALS patient classification [36,90]. Also, related literature supports that the “missing heritability” in genetic traits could be uncovered and explained to a significant degree by a network of gene-gene interactions which is not taken into account in the methods typically used to estimate the proportion of heritability that is missing [77,144,159]. Even though, the prediction of novel disease-specific gene-gene interactions poses, as described in previous chapters, a greater number of challenges than single-marker GWAS, it offers the potential to understand the heritability and genetic architecture of complex traits like ALS disease in greater depth. These challenges could be faced using machine learning approaches.

Machine Learning is a rapidly evolving field that has great potential in helping us to understand the complexity of ALS genomics, and how this relates to molecular pathways. However, further advances are needed in GWAS machine learning approaches in order to fully uncover the underlying mechanisms of this deadly disease which ultimately may lead us to successful personalized disease and drug-targeting prediction approaches.

## Figures and Tables

**Table 1:** Details of the feature selection approaches that have been followed by each study prior to the machine learning experiments. The “Data/Instances” column summarises the primary number of instances before further filtering, and details about the datasets that have been used by each study. The “Features” column refers to the initial number of features in each dataset before feature selection and after quality control (the latter being applicable only in studies using genotype data). The “Epistasis” column indicates whether the respective study has incorporated epistatic events (i.e., multi-locus interactions) as a feature selection method. The “Regulatory Elements” column describes studies that have filtered their initial dataset by including only non-coding regulatory regions. Studies that have used prior ALS-related information (e.g., already known ALS-associated SNPs/genes, known functional information, filtering based on an ALS versus Control genotype-phenotype association analysis *p*-value etc.) in order to select and reduce their initial instance and feature space are indicated under “ALS-linked knowledge”. Lastly, we indicate machine learning methods that were used to select only highly informative features based on specific criteria. ML: Machine Learning, SNP: Single Nucleotide Polymorphism, MDR: Multifactor Dimensionality Reduction, CNN: Convolutional Neural Network, PPIs: Protein- Protein Interactions, DHS: DNase I hypersensitive sites, TFBS: Transcription Factor Binding Sites, PCA: Principal Component Analysis, t-SNE: t-distributed Stochastic Neighboring Embedding, UMAP: Uniform Manifold Approximation and Projection.

Study	Data/Instances	Features	Genomic Structure	Epistasis	Cis-Regulatory Elements	ALS-Linked Knowledge	ML Methods
Vitsios et al. [103]	18,626 coding genes (label:positive/unlabelled)	1,249 gene-annotations: generic, disease- and tissue -specific features	No	No	No	Yes	PCA, t-SNE, UMAP
Yousefian et al. [104]	8,697,640 SNP *p*-values of 14,791 ALS cases and 26,898 controls [36]	2,252 functional features: DHS mapping data, histone modifications, target gene functions, and TFBS	Yes	No	Yes	Yes	None
Bean et al. [105]	ALS-linked gene lists: DisGeNet: 101 genes ALSoD: 126 genes, ClinVar: 44 genes, Manual list: 40 genes Union: 199 genes	PPIs, disease-gene associations and functional annotations	No	No	No	Yes	None
Yin et al. [90]	4511 cases and 7397 controls [16]	823,504 SNPs from 7, 9, 17 and 22 chromosomes	Yes	No	Yes	Yes	CNN
Kim et al. [91]	SNP pairwise interactions	550,000 SNPs of 276 cases/271 controls and 211 cases/211 controls [76,106]	No	Yes	No	Yes	MDR
Greene et al. [107]	SNP pairwise interactions	210,382 SNPs of 276 cases/271 controls and 211 cases/211 controls [76,106]	No	Yes	No	No	MDR
Sha et al. [108]	SNP pairwise interactions	555,352 SNPs of 276 cases/271 controls [76]	No	No	No	Yes	None

**Table 2:** The biological hypotheses incorporated to the experimental design and main focuses of the collected studies.

	Genomic Structure	Epistasis	Cis-Regulatory Elements	ALS-Linked Knowledge	Functional Annotations
Vitsios et al. [103]	No	No	No	Yes	Yes
Yousefian et al. [104]	Yes	No	Yes	Yes	Yes
Bean et al. [105]	No	No	No	Yes	Yes
Yin et al. [90]	Yes	Yes	Yes	No	No
Kim et al. [91]	No	Yes	Yes	Yes	Yes
Greene et al. [107]	No	Yes	No	No	No
Sha et al. [108]	No	Yes	No	Yes	No

**Table 3:** The machine learning approaches followed by each study. Acc: Accuracy, SVM: Support Vector Machines, LR: Logistic Regression, RF: Random Forest, DNN: Deep Neural Network, SVC: Support Vector Classifier (SVC), CNN: Convolutional Neural Network, AUC: Area under the receiver operator characteristic curve, CSM: Combinatorial Searching Method, Chr: Chromosome.

Study	Machine Learning Models	Model Assessment	Performance
Vitsios et al. [103]	Stochastic Semi-supervised Learning: Stacking, DNN, Gradient Boosting, RF, SVC, XGBoost, ExtraTrees Classifiers	10-fold cross validation	Stacking: Avg AUC: 0.767, DNN: Avg AUC: 0.774, Gradient Boosting: Avg AUC: 0.79, RF: Avg AUC: 0.798, SVC: Avg AUC: 0.801, XGBoost: Avg AUC: 0.805, ExtraTrees: Avg AUC: 0.814
Yousefian et al. [104]	Convolutional Neural Network	Autoencoder pre-training, Chr 1-10: training set, Chr 11–14: testing set and Chr 15–22: validation set	CNN: AUC: 0.96 F1-score: 0.83
Bean et al. [105]	Knowledge graph edge prediction model [116]	5-fold cross validation	Fold-Change Enrichment and random guess baseline: ALSoD: 23.33 (23.53), ClinVar: 30.05 (15.64), DisGeNet: 55.90 (81.66), Manual: 84.54 (13.27), Union: 8.92 (4.28)
Yin et al. [90]	Deep Neural Network (ALS-Net), Logistic Regression, SVM, Random Forest and Adaboost	9-fold cross validation	ALS-Net: Acc: 0.769 F1-score: 0.797 LR: Acc: 0.739 F1-score:0.728 SVM: Acc: 0.725 F1-score:0.694 RM: Acc: 0.596 F1-score:0.381 Adaboost: Acc: 0.661 F1-score:0.625 (+PromoterCNN and all 4 chromosomes combined)
Kim et al. [91]	Multifactor dimensionality reduction; using a naïve Bayes classifier	1000 permutation tests	Critical Acc: 0.629 and 0.640 (replication dataset)
Greene et al. [107]	Multifactor dimensionality reduction	1000 permutation tests	Best SNP pairwise model: Acc: 0.6551 and 0.5821 (replication dataset); with *p* < 0.048 and *p* < 0.021 (replication dataset)
Sha et al. [108]	Two-locus probabilistic models, Multifactor dimensionality reduction, Combinatorial Searching Method	1000 permutation tests	Two-locus models: rs4363506-rs3733242: *p* = 0.032 rs4363506-rs16984239: *p* = 0.042 MDR model: rs4363506-rs12680546: *p* = 0.156 CSM model: rs4363506-rs12680546: *p* = 0.2
